# On the validity of area-based income measures to proxy household income

**DOI:** 10.1186/1472-6963-8-79

**Published:** 2008-04-10

**Authors:** Gillian E Hanley, Steve Morgan

**Affiliations:** 1 Centre for Health Services and Policy Research, University of British Columbia, Vancouver, BC Canada; 2 Department of Health Care and Epidemiology, University of British Columbia, Vancouver, BC Canada

## Abstract

**Background:**

This paper assesses the agreement between household-level income data and an area-based income measure, and whether or not discrepancies create meaningful differences when applied in regression equations estimating total household prescription drug expenditures.

**Methods:**

Using administrative data files for the population of BC, Canada, we calculate income deciles from both area-based census data and Canada Revenue Agency validated household-level data. These deciles are then compared for misclassification. Spearman's correlation, kappa coefficients and weighted kappa coefficients are all calculated. We then assess the validity of using the area-based income measure as a proxy for household income in regression equations explaining socio-economic inequalities in total prescription drug expenditures.

**Results:**

The variability between household-level income and area-based income is large. Only 37% of households are classified by area-based measures to be within one decile of the classification based on household-level incomes. Statistical evidence of the disagreement between income measures also indicates substantial misclassification, with Spearman's correlations, kappa coefficients and weighted kappa coefficients all indicating little agreement. The regression results show that the size of the coefficients changes considerably when area-based measures are used instead of household-level measures, and that use of area-based measures smooths out important variation across the income distribution.

**Conclusion:**

These results suggest that, in some contexts, the choice of area-based versus household-level income can drive conclusions in an important way. Access to reliable household-level income/socio-economic data such as the tax-validated data used in this study would unambiguously improve health research and therefore the evidence on which health and social policy would ideally rest.

## Background

Measures of income are often central to health and health policy research. Among many potential implications, income can be a non-medical determinant of health [1-3] an enabling factor for access to care[4], or a consideration when judging equity of policies and programs[5]. As important as this variable may be, it is often difficult for health and health policy researchers to obtain reliable, individual-level income information for the populations they study. In the absence of individual-level income data, investigators often supplement health research datasets with group-based measures such as area-based average income constructed from national census data[6]. Such measures are used as proxy for individual-level income data on the assumption that household incomes will be reasonably homogeneous within small enough residential areas. If, however, there is significant heterogeneity in the areas used, then the aggregate measures can result in ecological fallacy–when an association observed between variables at an aggregate level does not represent the association that exists at an individual level[7].

Prior studies have investigated misclassification of income and other socio-economic variables by comparing individual versus area-level survey responses for small samples of the population[8,9] and by comparing survey-based measures for different sized census areas[10,11]. Using a unique dataset that contains validated household income data for approximately 78% of the population of British Columbia (BC), we investigate the level of misclassification that can occur when census-defined, area-based income is used as a proxy for an individual's actual household-level income. As the question of most interest to researchers concerns how well aggregate variables perform when they are entered in health outcomes equations, we then assess the sensitivity of the analysis of health related inequities in total prescription drug costs to whether income is measured as an area-based variable or an household-level variable.

## Methods

### Data

Our primary datasets are administrative files for the provincially administered, universal public medical and hospital health insurance program, Medical Services Plan (MSP) of BC. This program covers virtually all 4.2 million residents of BC, excluding only those residents covered by federal health insurance programs (collectively about 4% of the population). We restrict our attention to households for which one or more member resided in BC for at least 275 days per year from 2001 to 2004, inclusive.

Household income was obtained from the 2004 registration files for provincially administered, universal public pharmaceutical insurance program, BC PharmaCare. In addition to programs for social assistance recipients and other select populations, BC PharmaCare began offering income-based public drug coverage to all residents of the province in May 2003. Terms such as deductibles and co-insurance are based on household income, with more generous but still income-based coverage offered to senior citizens (residents aged 65 and older). For all households that registered to receive coverage, the BC Ministry of Health obtains net, pre-tax income information from the Canada Revenue Agency. Because of differences in coverage offered and average needs, 95% of households with one or more senior member were registered for Fair PharmaCare in 2004 whereas only 73% of non-senior households were registered.

The area-based income variables used in this study are based on linking MSP registry postal codes to average household income in the area as recorded in the 2001 Census. Statistics Canada collates average household income and composition for over 7,000 Census Dissemination Areas comprised of 400 to 700 persons. For research purposes, these areas are sorted by income and aggregated into 1,000 strata. Income strata contain an average of 1,700 households, with some variation due to variations in populations by postal code. Both the household level and area-based income variables are based on the same income concept, gross income prior to any deductions.

Total individual expenditures on prescription drugs were obtained from BC PharmaNet. BC PharmaNet is an administrative dataset in which every prescription dispensed in the province must be entered by law–it is designed to support drug dispensing, drug monitoring and claims processing. These individual expenditures were aggregated at the household level according to registration files for the MSP program to create a variable indicating total household spending on prescription drugs.

The research data were extracted for this study from the British Columbia Linked Health Database and the BC PharmaNet database with permission of the BC Ministry of Health and the College of Pharmacists of BC. Ethics approval was obtained from the Behavioural Research Ethics Board at the University of British Columbia.

### Statistical methods

The household-specific and area-based income measures were each aggregated into deciles (ordered from lowest to highest income). We assess the discrepancy between the two measures using the CRA validated, household-specific incomes as the standard. We calculated the Spearman's rank correlations of the various income measures, and both the kappa and weighted kappa to measure the degree of non-random agreement and partial agreement between the measures.

We proceed to examine whether the choice of income measure has an impact on how pharmaceutical expenditures are distributed by income status. We begin by examining the distribution of prescription drug expenditures by income deciles, where the deciles are defined according to household-level income then according to neighbourhood level income. As measurement error is accommodated more easily in regression analysis than in descriptive analysis, we also include a series of dummy variables for both versions of the income variable in an OLS regression in order to determine whether both area-based income and household income generate meaningfully different results when applied in a research context. We perform regressions of income on total drug expenditures with and without covariates controlling for the presence of one or more seniors in the household as well as household size. Through the comparison of coefficients between household-level income variables and area-level income variables, one can reach some conclusions about the appropriateness of substituting an area-based measure for a missing household-level variable in a regression equation. By including regressions with and without covariates, we can determine whether multivariate models influence the discrepancy between area-based and household-level variables.

## Results

A total of 1.74 million households were registered for MSP and had valid postal codes for linkage with area-based income strata. This cohort accounts for 95% of the total population in the province. Of these households, 1.36 million were registered for the Fair PharmaCare program. Cross-tabulations of the household-level and area-based income measures are shown in Table 1, where NR indicates the percentage of households in each area-based decile who were not registered for the Fair PharmaCare program at the time of data collection. This table confirms that rates of participation with the income-based program are lower in higher income neighbourhoods. This concentration of low incomes for the household-level income variable is because the registration for income-based drug coverage involves a degree of self-selection bias. To adjust for this, our tables below present a "best case" scenario wherein all non-registered households are assigned a hypothetical household-level income variable that is identical to their area-level income.

**Table 1:** Entire BC population, 2003. Agreement between household-level validated income deciles and area-based income deciles

Household-level validated income decile													
		1	2	3	4	5	6	7	8	9	10	NR	total

Area-based income decile	1	**20.1**	13.0	13.1	10.8	7.7	5.7	5.0	3.6	2.6	1.7	16.6	100
	2	10.8	**10.5**	10.7	10.4	9.4	8.2	7.6	5.9	4.6	2.7	19.2	100
	3	9.1	9.2	**9.7**	9.7	9.5	8.7	8.1	6.8	5.7	3.5	20.0	100
	4	7.8	8.2	8.9	**9.2**	9.2	8.8	8.5	7.7	6.7	4.3	20.8	100
	5	6.8	7.6	7.7	8.3	**8.6**	8.7	8.6	8.5	7.8	5.4	22.1	100
	6	5.7	7.0	7.1	7.5	8.2	**8.6**	8.7	8.8	8.9	6.8	22.8	100
	7	4.9	6.5	6.3	6.8	7.7	8.4	**8.7**	9.3	9.7	8.6	23.2	100
	8	4.7	6.0	5.6	6.0	6.8	7.9	8.5	**9.6**	10.8	11.0	23.2	100
	9	4.0	5.4	5.0	5.2	6.0	7.4	8.1	9.6	**11.1**	14.1	24.1	100
	10	4.2	4.8	4.2	4.2	4.9	5.9	6.5	8.3	10.2	**20.1**	26.6	100

Note: NR indicates individuals who were not registered for Fair PharmaCare at the time of data collection.

Table 2 shows the level of discrepancy between the household-level and area-based income measures. The area-based measures classify 15.6% of senior households and 14.9% of non-senior households as being within the same income-decile as is determined by tax-reported household income. Approximately a third of non-senior households and two fifths of senior households are classified by area-based measures to be within one decile of the classification based on household-level incomes. In the "best-case" scenario, just over half of non-seniors and approximately 43% of seniors are within one decile of their household-level income.

**Table 2:** Percentage of discrepancy by decile between area-based and household-level income measures

Area-based Measure	Group	None	One	Two	Three	Four	Five	Six	Seven	Eight	Nine
Actual household-level income	All	14.9	22.5	18.5	14.5	10.8	7.7	5.3	3.3	1.8	0.7
	Non-seniors	14.9	21.8	18.2	14.7	11.2	7.9	5.4	3.4	1.8	0.7
	Seniors	15.6	23.9	19.1	14.4	10.4	7.2	4.7	2.7	1.4	0.6

Best-case scenario	All	33.4	18.3	14.4	11.4	8.3	5.4	4.1	2.6	1.4	0.7
	Non-seniors	37.8	16.0	13.3	10.7	8.2	5.8	3.9	2.5	1.3	0.5
	Seniors	20.2	22.7	18.0	13.6	9.8	6.8	4.4	2.6	1.3	0.6

Note: In Best-case scenario, non-registered households are assigned a hypothetical household-level income decile that is identical to their area-based measure.

Statistical evidence of the disagreement between income measures can be found in Table 3. The Spearman's correlations between the actual household-level income and area-based measures are always less than 0.40, suggesting little agreement. The kappa coefficient of non-random, complete agreement never exceeds 0.31 indicating very little complete agreement between area-based and actual household-level deciles even under the assumption of perfect correlation between area-based and household-level measures for all non-registrants. Again, when examining the weighted kappa coefficients, incorporating partial agreement, we see that they never exceed 0.5, even in the best-case scenario.

**Table 3:** Spearman's correlation, Kappa and weighted Kappa coefficients for the association between the area-based income measures and the household income measure

Area-based measure	Group	r_s_	Kappa	Weighted Kappa
Actual household-level income	All	0.322	0.055	0.322
	Non-seniors	0.316	0.054	0.315
	Seniors	0.382	0.060	0.382

Best-case Scenario (including non-registrants)	All	0.469	0.260	0.469
	Non-seniors	0.497	0.309	0.496
	Seniors	0.420	0.113	0.420

Note: r_s _= Spearman's correlation coefficient.

In Best-Case Scenario, non-registered households are assigned a hypothetical household-level income decile that is identical to their area-based measure.

To examine whether these discrepancies result in any meaningful differences in an applied research context, we start by examining the distribution of total prescription drug expenditures by income deciles stratified by senior and non-senior households, first using household-level CRA validated income and then using aggregate neighbourhood level income (Table 4). Table 4 indicates that total prescription drug expenditures appear more equally distributed when we rank households by neighbourhood income than by household-level income, suggesting that neighbourhood level income masks variation in the underlying household-level income variable.

**Table 4:** Total drug costs by income decile

Entire BC population			Non-senior population		Senior population	
	Mean total drug costs	Percent of total drug costs	Mean total drug costs	Percent of total drug costs	Mean total drug costs	Percent of total drug costs

Deciles measured by CRA validated income						

1 ($900–3,750)	743.27	10.39	826.46	15.24	894.61	6.99
2 ($3750–12,000)	615.16	8.60	456.26	8.41	942.67	7.37
3 ($12,000–16,000)	605.19	8.46	332.54	6.13	1059.92	8.28
4 ($16,000–20,000)	681.31	9.53	334.55	6.17	1178.95	9.21
5 ($20,000–29,375)	534.48	7.47	358.74	6.61	1280.50	10.01
6 ($29,375–39,584)	653.91	9.14	411.01	7.58	1352.22	10.57
7 ($29,584–51,250)	705.15	9.86	503.90	9.29	1420.86	11.11
8 ($51,250–67,917)	821.07	11.48	654.20	12.06	1499.01	11.72
9 ($67,917–91,667)	881.27	12.32	741.34	13.67	1565.45	12.24
10 ($91,667–475,000)	912.01	12.75	804.41	14.83	1599.72	12.50
		100		100		100

Deciles measured by neighbourhood income						

1 ($4,200–20,100)	726.63	10.16	615.32	11.35	1109.80	8.67
2 ($20,100–23,800)	653.33	9.13	499.71	9.21	1189.72	9.30
3 ($23,800–26,500)	697.09	9.75	511.38	9.43	1245.68	9.74
4 ($26,500–29,00)	700.24	9.79	510.90	9.42	1273.95	9.96
5 ($29,00–31,300)	707.28	9.89	523.97	9.66	1271.18	9.94
6 $31,300–33,900)	711.68	9.95	531.67	9.80	1309.01	10.23
7 ($33,900–36,900)	720.97	10.08	540.98	9.97	1323.70	10.35
8 ($36,900–41,300)	722.07	10.09	545.90	10.07	1343.11	10.50
9 ($41,300–48,900)	728.69	10.19	554.05	10.22	1349.44	10.55
10 ($48,900–310,900)	784.83	10.97	589.86	10.87	1378.30	10.77
		100		100		100

Note: All numbers are based on the best-case scenario in which all non-registered households are assigned a hypothetical individual-level income decile that is identical to their area-based measure.

In Table 5 we estimate the effect of household income on total prescription drug expenditures by using both household-level income and neighbourhood level income in separate regressions. The dummy variable for the highest income decile was not included in the regression; thus, the coefficients can be interpreted as the difference in total prescription drug costs between each income decile and the highest income decile. The regression results also reflect the pattern noted in Table 4. While the signs never differ, the household-level variables pick up a substantially larger coefficient than the corresponding neighbourhood-level variable. This again suggests that the neighbourhood-level variables are smoothing the distribution of total prescription drug expenditures across income deciles. While the coefficients on income deciles differ substantially between the two models, it is interesting to note that the coefficients on presence of seniors and household size do not. Both coefficients are in the same direction and are of the same magnitude indicating that the difference in income variable does not have a large effect on other coefficients in the model. The model based on household-level income also reports a higher adjusted R[2] statistic than that using the area-based measure, indicating that the goodness of fit is higher in the regression using household-level variables. We also find that the inclusion of covariates in the model does not attenuate the bias between the variables substantially (Table 5).

**Table 5:** Results for the regression of dummy variables indicating income decile against total drug costs

Explanatory Variables	Household income	Neighborhood Income	Household income (without covariates)	Neighborhood Income (without covariates)
Income decile 1	-169.00 (-30.27)	-59.162 (-10.57)	-68.22 (-9.87)	-14.57 (-2.65)
Income decile 2	-297.08 (-53.20)	-128.26 (-22.92)	-405.50 (-58.67)	-96.59 (-17.61)
Income decile 3	-305.05 (-54.63)	-91.67 (-16.38)	-289.43 (-41.87)	-80.34 (-14.65)
Income decile 4	-232.39 (-41.62)	-85.20 (-15.22)	-179.73 (-26.00)	-72.91 (-13.29)
Income decile 5	-378.51 (-67.78)	-77.08 (-13.77)	-41.74 (-6.04)	-67.03 (-12.22)
Income decile 6	-257.56 (-46.13)	-74.33 (-13.28)	-40.48 (-5.85)	-54.49 (-9.94)
Income decile 7	-207.80 (-37.21)	-64.83 (-11.58)	-34.68 (-5.02)	-44.99 (-8.21)
Income decile 8	-89.99 (-16.12)	-62.60 (-11.18)	-53.00 (7.67)	-39.09 (-7.13)
Income decile 9	-32.60 (-5.84)	-57.38 (-10.25)	-14.63 (-2.12)	-34.05 (-6.21)
Presence of seniors	787.81 (250.77)	790.37 (267.87)	Not included	Not included
Household size	105.43 (89.26)	104.65 (92.14)	Not included	Not included
Adjusted R^2^	0.101	0.06	0.01	0.01

Note: T statistics are in parentheses. All coefficients are significant at 95% confidence interval.

## Discussion

We found a sufficient level of discrepancy between the area-based and household-level income measures. Using validated household income as the standard, area-based measures misclassified the income decile for eighty-five percent or more of the households in the data. We also found that these discrepancies did affect the size of coefficients in regression analyses, suggesting that very different conclusions can be reached regarding the 'same' issue depending on which income variable we use. Thus, these results indicate that, at least in some contexts, the choice of neighbourhood versus household income can drive conclusions in an important way. Our results are consistent with a large amount of work indicating substantial discrepancy between area-based and household SES measures[2,6,8,10].

There are also a couple of important caveats. The first is that our study did not examine the inclusion of income as simply one of several control variables, but rather only looked at the difference between household-level and area-level income when applied as the primary variable of interest. Thus, results cannot be extended to the use of income as a control in much larger regression equations. Second, these results are not meant to suggest that the use of neighbourhood income is inferior in all contexts. An author particularly concerned with measuring permanent income free of yearly fluctuations may find that neighbourhood income provides a better measure. When measuring access to health care, it might also be true that low-income families living in high-income neighbourhoods have better access to care than other similar low-income families simply because of where they live. Thus, an argument could be made for including both measures in this type of work.

While the level of agreement between area-based and household-level SES measures has frequently been studied, our work adds to the knowledge base for several reasons. It encompasses a larger number of Canadians, a sample of 78% of all households in British Columbia, of which 95% of all senior households are analyzed. Also, while other studies have tended to compare area-based measures to household-level survey data[6,8,9] or have compared two or more different sized area-based measures[10,11] we have used highly reliable household-level income data validated with the Canada Revenue Agency. Therefore, we have been able to avoid all self-reporting bias, we have a great deal of confidence in our household-level income variable, and we have been able to analyze almost an entire population of a Canadian province.

## Conclusion

While many authors have argued that household-level income should be used whenever possible, census-based aggregate measures will continue to be necessary for health research until household-level data become more readily available. Two suggestions can be made based on these research results. The first is that researchers should be cautious when interpreting the results of studies using aggregate measures as proxies for individual and household income. Area-based measures are approximations that are best suited to investigating major differences in incomes (e.g., differences of two or more quintiles) or to studying context in which someone lives rather than their specific income. The second suggestion is perhaps obvious to researchers but important for governments and statistical agencies to fully understand: access to reliable individual-level income/socio-economic data, as well as the neighbourhood level income data that is currently available, would unambiguously improve health research and therefore the evidence on which health and social policy would ideally rest.

## Competing interests

The author(s) declare that they have no competing interests.

## Authors' contributions

GH participated in conception of the study and study design, performed the statistical analysis and drafted the manuscript. SM participated in conception of the study and study design and participated in drafting the manuscript. Both authors read and approved the final manuscript.

## Pre-publication history

The pre-publication history for this paper can be accessed here:


